# Is the clinical phenotype impact the prognosis in dementia with Lewy bodies?

**DOI:** 10.1186/s13195-023-01305-7

**Published:** 2023-10-11

**Authors:** Clément Aveneau, David Wallon, Bertrand Degos, Alexandre Obadia, Claire Hourregue, Sarah Benisty, Béatrice Garcin, Julien Dumurgier, Claire Paquet

**Affiliations:** 1 Cognitive Neurology Center, Université Paris Cité, Lariboisière Fernand Widal Hospital, Assistance PubliqueHôpitaux de Paris, Paris, France; 2 INSERM U1144, Therapeutic Optimization in Neuropsychopharmacology, Paris, France; 3 Department of Neurology and CNR-MAJ, 76000INSERM U1245, Normandy Center for Genomic and Personalized Medicine, Normandie University, UNIROUEN, CHU Rouen, Rouen, France; 4 Neurology Department, Avicenne Hospital, APHP, Hôpitaux, Universitaires de Paris-Seine Saint Denis (HUPSSD), Sorbonne Paris Nord, Réseau NS-PARK/FCRIN, Bobigny, France; 5 Neurology Department, Fondation Adolphe de Rothschild, Paris, France

**Keywords:** Dementia with Lewy body, Prognosis, Clinical subtypes, Onset, Prodromal

## Abstract

**Introduction:**

The first predominant clinical symptoms of dementia with Lewy bodies (DLB) are highly variable; however, the prognosis based on initial predominant symptoms remains poorly understood.

**Methods:**

Multicenter retrospective study in 4 French expert neurological centers. Patients were categorized in 3 groups according to their first more predominant symptoms: cognitive, psychiatric, or motor.

**Results:**

Analysis of 310 DLB patients. The mean age was 73.5 years old (SD 7.5) including 32.3% of women. The mean follow-up was 7.25 years (SD 3.6). We observed that the full clinical picture was more frequent in the motor group than in the cognitive group (*p* = 0.01); male gender and age at onset were associated with a significant excess risk of instantaneous mortality (*p* = 0.01).

**Conclusion:**

Initial symptoms may affect the clinical course of patients, but no significant difference in mortality was observed.

## Introduction

Despite being the second most prevalent form of neurodegenerative cognitive decline, dementia with Lewy bodies (DLB) is often under-diagnosed and under-researched, resulting in inadequate therapeutic management. Neuropathologically, DLB is characterized by neuronal inclusions containing aggregates of phosphorylated α-synuclein with varying distribution patterns, as well as frequent co-pathology with Alzheimer’s disease (AD) markers (β-amyloid and phosphorylated tau accumulation). This neuropathological variability contributes, at least in part, to the heterogeneity of clinical phenotypes [1, 2]. DLB patients experience a range of neuropsychiatric, cognitive, dysautonomic, sleep, and motor symptoms; however, not all patients exhibit every clinical sign or develop all symptoms during the disease progression. Additionally, the order of symptom occurrence and severity may differ among patients, resulting in a wide array of phenotypes and delayed diagnosis [3]. This clinical variability is widely recognized in everyday clinical practice; nevertheless, DLB clinical phenotypes have been scarcely investigated. To date, two studies have specifically compared the progression of various DLB one based on clinical symptoms [4] and the second on the brain MRI pattern [5]. In addition, one study has shown that patients presenting with prominent psychiatric symptoms may experience increased morbidity and nursing home placements frequency [6]. Understanding the relationship between DLB phenotype and prognosis can inform clinical decision-making and help tailor interventions to address the specific needs of individuals with DLB.

In this multicentric study, our objective was to investigate the impact of the phenotype categorized by the first more predominant symptoms (cognitive, motor, psychiatric) on the prognosis of DLB in terms of survival and functional independence. We examined data from patients with DLB attending two memory clinics and two Parkinson’s disease specialty centers in France. Patients were classified into three DLB subgroups based on the predominance of initial clinical symptoms: cognitive, motor, or psychiatric. By comparing the prognosis of these subgroups, we seek to provide valuable insights into the relationship between initial clinical presentation and long-term outcomes in DLB.

## Methods

### Study population

We performed a multicentric retrospective study including patients diagnosed with DLB between 2006 and 2021 and meeting McKeith’s criteria for probable DLB [7]. A subgroup of included population underwent a lumbar puncture for cerebrospinal fluid (CSF) biomarkers. In order to include various clinical phenotypes at onset, we performed the study in two specialized memory clinics and two centers specializing in Parkinson’s disease (PD). Patients were categorized as cognitive, psychiatric, or motor predominant onset, based on the descriptions available in the medical records. We defined a “cognitive onset” when symptoms began with a cognitive complaint, cognitive impairment, or fluctuation of alertness or confusion; a “psychiatric onset” when symptoms began with hallucinations/illusions/delusions or a mood disorder occurring after the age of 50 and without any trigger factor (bereavement, trauma…); and a “motor onset” when symptoms began with a parkinsonian syndrome. In patients with a predominance of motor symptoms at onset, differentiation from (PD) has been made according to the “1-year rule,” which indicates a diagnosis of DLB when cognitive dysfunction precedes or occurs less than 1 year after the onset of motor symptoms. We defined the presence of a “full clinical picture” when patients had a combination of motor, cognitive, and psychiatric symptoms. The study was approved by the Bichat Hospital Ethics Committee of Paris Diderot University.

### Outcome

The primary outcome of this study was the time from diagnosis to either death or poor prognosis, defined as the need for institutionalization, home helpers or death.

This allowed us to investigate the impact of the initial clinical presentation on the prognosis of LBD in terms of survival and autonomy.

### Covariates

The following data were collected: demographic data (age, sex, education level), medical history of the patients and his/her family, duration of the follow-up, year and type of the first (or predominant first) symptoms, delay between first symptoms and the complete clinical picture, date of death, entry into an institution or need for external assistance for the acts of daily life, transversal and longitudinal MMSE, CSF AD biomarkers.

### Statistical analysis

We used Kruskal–Wallis and chi-square models to examine the clinical characteristics of patients, while Kaplan–Meier curves, log-rank tests, and Cox models were used to investigate the relationship between clinical onset and patient outcomes, specifically mortality and poor prognosis. Poor prognosis was defined as a composite criterion that included mortality, entry into institutional care, or a requirement for external assistance with daily living activities. We conducted multivariate analyses, accounting for age at symptom onset, sex, and recruitment center in the first model (Cox1), and further incorporating cardiovascular risk factors in the second model (Cox2). A *p*-value less than 0.05 (two-tailed) was considered statistically significant. We performed analyses and generated graphics using R studio (version 4.0.3).

## Results

### Demographics

Data from 310 patients were collected from four tertiary centers and analyzed. Table 1 provides a summary of the whole study cohort characteristics. A total of 128 patients underwent lumbar punctures to assess Alzheimer’s biomarkers, which we were able to obtain in 126 of them. No significant differences were observed between the groups concerning demographic characteristics, cardiovascular risk factors, initial cognitive scores, and AD CSF biomarkers. The average age at clinical onset was 69.8 years, while the mean age at diagnosis was 73.5 years, with a male predominance of 67.7% (210 out of 310). The most frequently reported initial symptoms were cognitive impairment (60.6%), followed by motor disorders (27.1%), and finally, psychiatric disturbances (12.3%). Notable differences in the prevalence of clinical symptoms such as parkinsonian syndrome (*p* < 0.01), visual hallucinations (*p* < 0.001), and the occurrence of falls (*p* < 0.01) were found between the groups. Patients with motor onset exhibited parkinsonian syndrome and falls more frequently, whereas those with cognitive onset experienced visual hallucinations less often. We also observed significant differences in the proportion of performed FDG PET scan and polysomnography that are increased in the psychiatric group, and DAT scan results are significantly more frequently abnormal in the motor group. In the same line, some treatments are differently prescribed according to the group (Table 1).

**Table 1:**
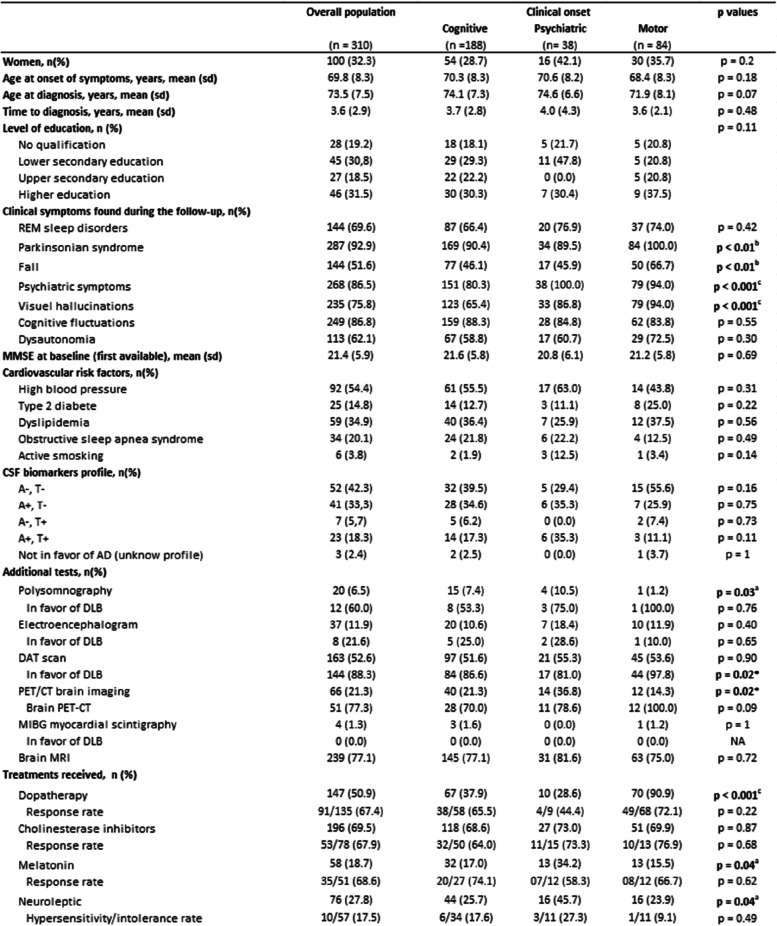
Patient sample characteristics

Data are represented as *n* (%) or mean (SD). *p* value for comparison of multiples qualitative data have been calculated using a chi-square test, and *p* values for comparison of multiples quantitative data have been calculated using a Kruskal–Wallis test

^a^*p* value < 0.05

^b^*p* value < 0.01

^c^*p* value < 0.001

### Data on mortality

The average follow-up duration in the study was 7.25 years (SD 3.6), spanning from the first symptoms to death or the end of the follow-up period. During this time, 40% (124 patients) of the participants passed away. Patient characteristics, based on whether they experienced death, are summarized in Table 2.

**Table 2:**
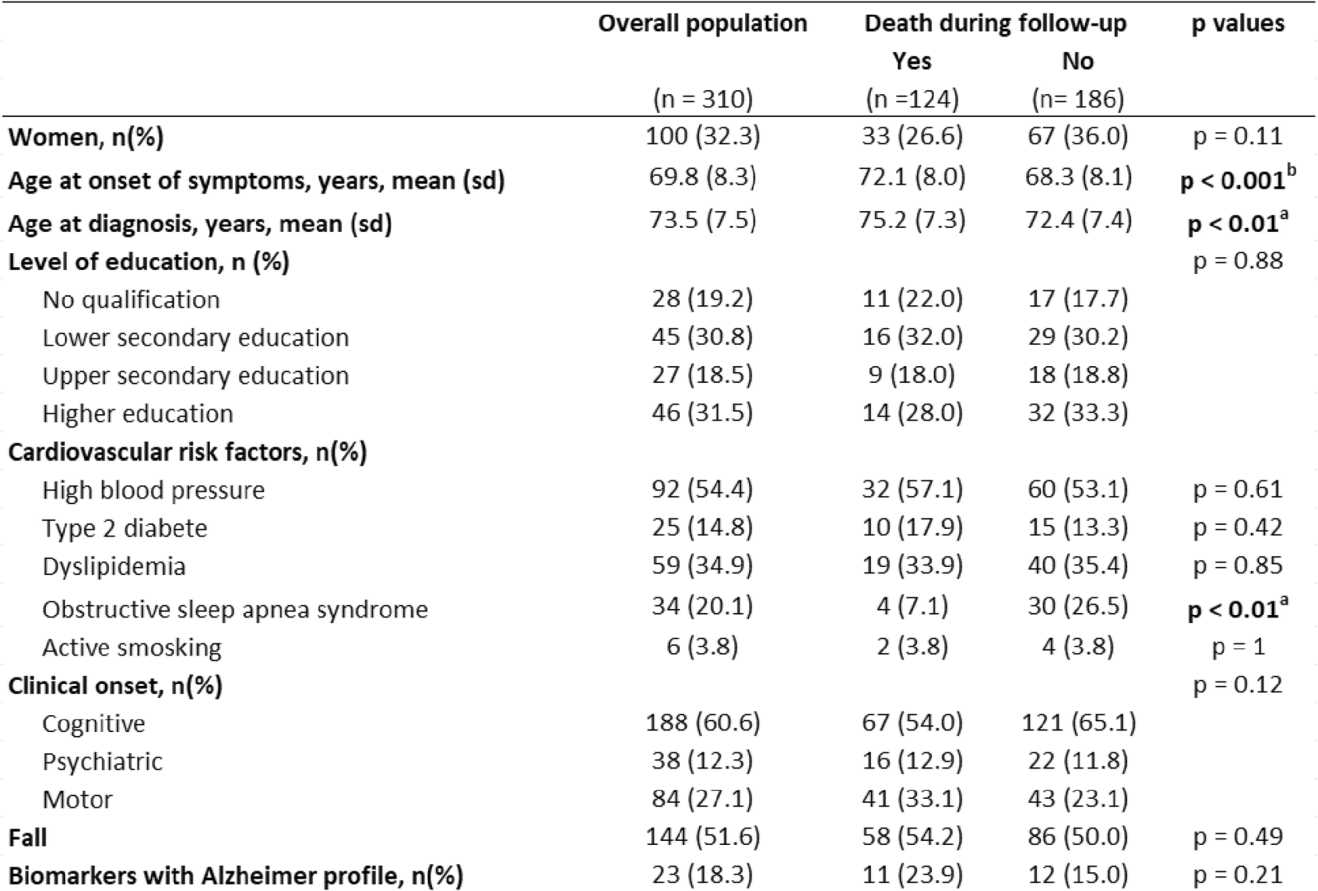
Characteristics of the population according to the occurrence of a death during follow-up

Data are represented as *n* (%) or mean (SD). *p* value for comparison of multiples qualitative data have been calculated using a chi-square test, and *p* values for comparison of multiples quantitative data have been calculated using a Kruskal–Wallis test

^a^*p* value < 0.01

^b^*p* value < 0.001

**Fig. 1:**
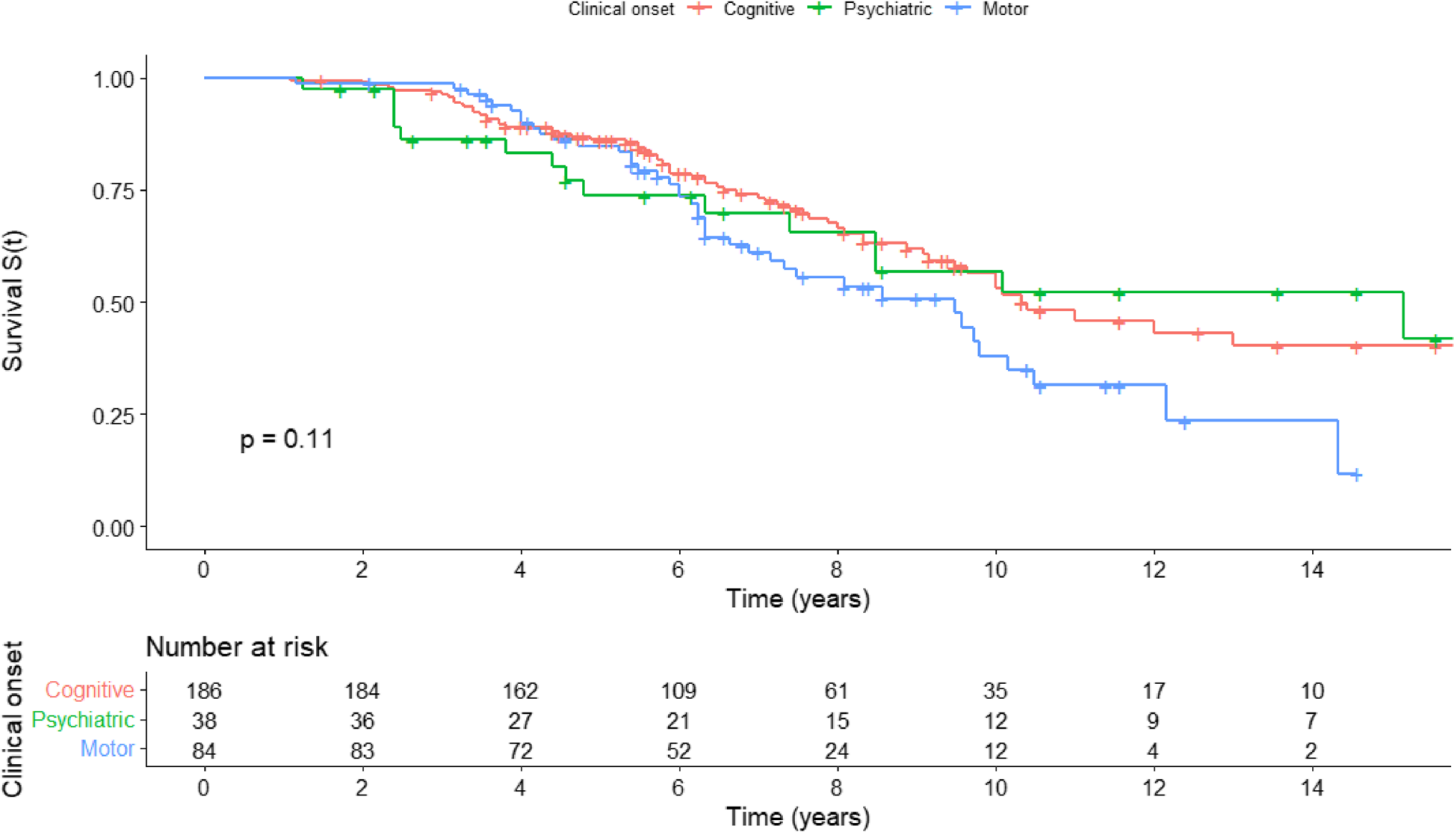
Kaplan–Meier survival curve (death) according to clinical entry points. Survival curve according to the Kaplan–Meier method for patients with motor (blue line), cognitive (red line), or psychiatric (green line) predominant clinical onset. The numbers below the curve indicate the number of patients at risk at each time point. Differences between the two groups were evaluated using a log-rank test (*p* = 0.11). Data were censored at the date of last observation (crosses) or death

In the whole study cohort, the calculated median survival (until death) was 10.0 years. The median survival for the cognitive group was 10.3 years, 9.5 years for the motor group, and 15.2 years for the psychiatric group. Figure 1 illustrates the Kaplan–Meier survival curve as a function of the initial predominant clinical symptoms. Upon visually analyzing the curve, a faster decline in the survival curve of subjects with motor clinical onset is observed compared to the other two dementia with Lewy bodies (DLB) clinical phenotypes from 6 years of follow-up. The evolution of these other phenotypes appears to be similar over time. No significant difference was detected using the log-rank test (*p* = 0.11).

Multivariate analyses employing a Cox model for mortality, poor prognosis, and full clinical picture are presented in Table 3. The first model includes age at onset, sex, and site of inclusion as explanatory variables, while the second model incorporates cardiovascular risk factors. Cox1 model for mortality shows a non-significantly higher instantaneous mortality risk in patients with motor or psychiatric onset compared to cognitive onset). Age at clinical onset (HR 1.10 [1.06–1.10], *p* < 0.001) and male gender (HR 1.70 [1.12–2.60], *p* = 0.01) were associated with a significant excess risk of instantaneous mortality. The second model yielded similar but non-significant results toward a higher instantaneous mortality risk in patients with motor or psychiatric onset compared to cognitive onset. Age at clinical onset and male gender continued to be associated with a higher risk of instantaneous mortality (HR 1.09 [1.06–1.12], *p* < 0.001 and HR 1.71 [1.12–2.62], *p* = 0.01). No significant risk difference was observed for the presence of hypertension (HR 0.91 [0.50–1.65], *p* = 0.76), diabetes (HR 1.69 [0.80–3.59], *p* = 0.17), dyslipidemia (HR 0.61 [0.32–1.14], *p* = 0.12), or active smoking (HR 1.59 [0.37–6.88], *p* = 0.54). A significant decrease in the risk of instantaneous mortality was found in patients with obstructive sleep apnea syndrome.

**Table 3:**
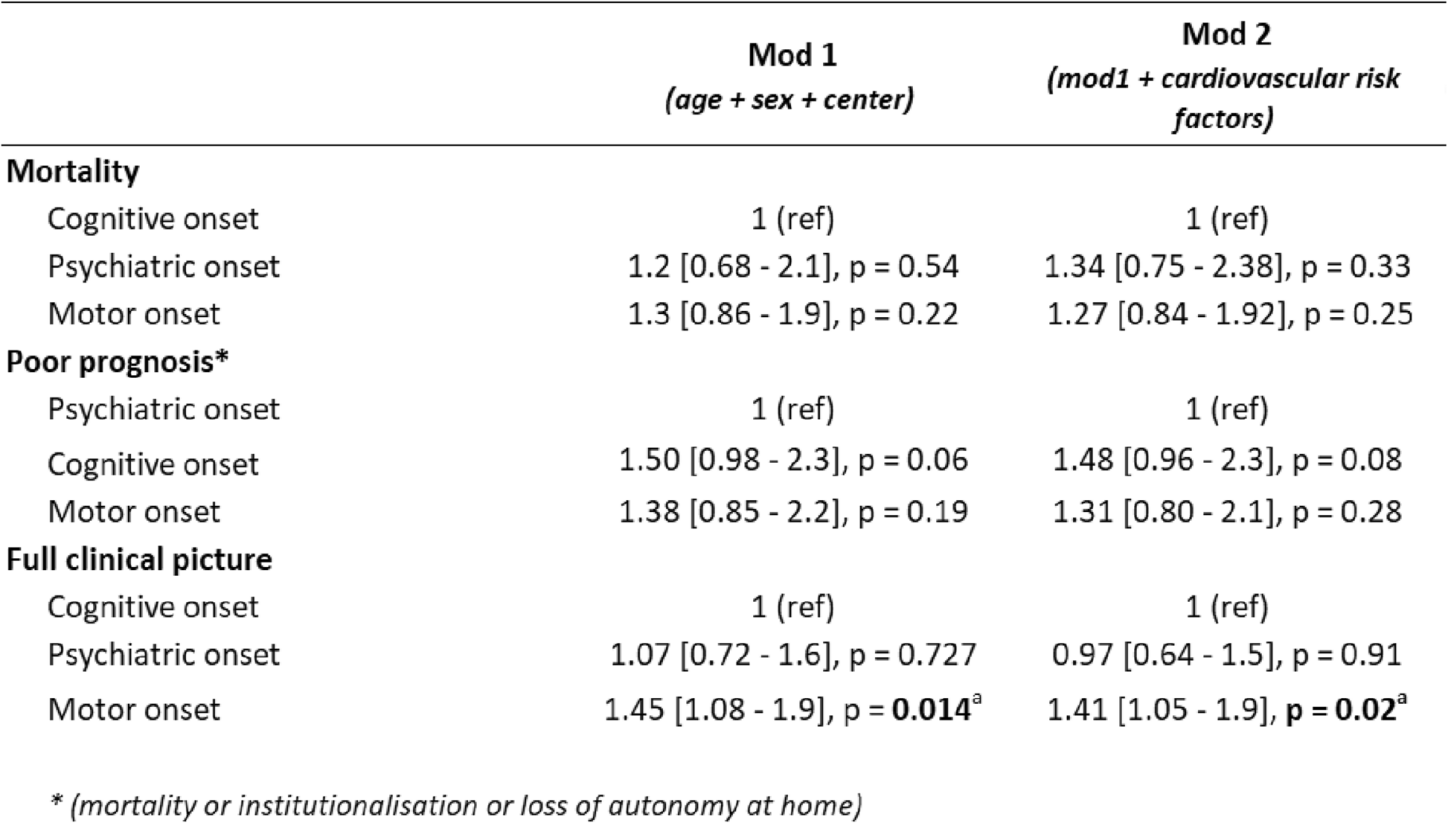
Cox analyses of mortality, pejorative evolution, and clinical course of patients

Comparison of the effects of clinical onset on mortality, poor prognosis event (death or loss of autonomy), and occurrence of a full clinical picture according to two Cox models. The first model (Cox1) includes age, sex, and neurological center of patient follow-up while the second model (Cox2) adds cardiovascular risk factors such as hypertension, diabetes, dyslipidemia, sleep apnea syndrome, and active smoking. Results are presented as hazard ratios (HR) with 95% confidence intervals. *P*-values are also provided for each factor in each model

^a^*p* value < 0.01

^b^*p* value < 0.001

### Data on loss of autonomy

In the whole study cohort, the median survival until a poor prognosis event or censorship is 5.7 years. This duration is 5.5 years in the cognitive group, 6.2 years in the motor group, and 6.3 years in the psychiatric group. The first Cox model revealed that age is significantly associated with a higher risk of poor prognosis (HR 1.08 [1.06–1.1], *p* < 0.001), while male gender is not in this model (HR 0.93 [0.70–1.2],* p* = 0.63). Additionally, there was a non-significant increased instantaneous risk of poor prognosis in the cognitive and motor groups compared to the psychiatric group (HR 1.50 [0.98–2.3], *p* = 0.06 and HR 1.38 [0.85–2.2], *p* = 0.19, respectively) as well as no excess risk of needing help at home (HR 1.83 [0.9–3.7], *p* = 0.1) or institutionalization (HR 1.06 [0.6–1.2], *p* = 0.9) for the cognitive onset group compared with the psychiatric onset group.

The second Cox model shows that a biological AD CSF biomarker profile, was significantly associated with a short-term risk of poor prognosis (HR 2.66 [1.46–34.8] *p* = 0.001) as well as active smoking (HR 3.38 [1.33–8.6] *p* = 0.01). We did not find any other significant associations regarding the phenotype or the comorbidities (hypertension, dyslipidemia, obstructive sleep apnea, diabetes).

### Clinical course

Out of the 310 patients included in the study, 246 (79.4%) exhibited a complete clinical picture (cognitive, psychiatric, and motor symptoms) during their follow-up, with an average delay of 3.41 years (SD 3.1). A complete clinical picture was observed in 133 of the 188 patients with a predominantly cognitive clinical onset (70.7%), with an average delay of 3.7 years (SD 2.9). In contrast, 79 of the 84 patients with a predominantly motor onset (94%) presented a complete clinical picture with an average delay of 2.6 years (SD 2.0), and 34 of the 38 patients with a psychiatric entry point (89.5%) exhibited a complete clinical picture with an average delay of 4.2 years (SD 4.8). Statistical comparisons between groups revealed a significantly increased risk of a full clinical picture in the motor predominant onset group compared to the cognitive predominant onset group (HR 1.45 [1.08–1.9] *p* = 0.014 in Cox1 model, HR 1.41 [1.05–1.9] *p* = 0.02 in Cox2 model). Additionally, there was a non-significant increase in risk compared to the psychiatric predominant onset group (HR 1.35 [0.86–2.1] *p* = 0.20 in Cox1 model, HR 1.45 [0.91–2.3] *p* = 0.12 in Cox2 model).

## Discussion

This large multicentric retrospective study aimed at exploring the clinical course and survival of patients in DLB according to their initial phenotype defined by the first predominant symptoms (cognitive, psychiatric, motor). We did not observe any significant difference in mortality or global prognosis between the 3 groups. We observed that age at clinical onset and male gender were associated with a higher risk of instantaneous mortality while sleep apnea syndrome was associated with a decreased risk. Due to the variable phenotypic characteristics between the three groups, we observed differences in clinical symptoms, complementary explorations, and treatments.

Regarding the previous studies that have explored the clinical subtype of DLB patients, Morena has categorized patients based on the initial clinical presentation while Inguanzo classified according to brain MRI analysis. Our study is different with larger cohort from a French region while Inguanzo performed an international imaging study and Morena performed a monocentric clinical study. The cohorts were also different (significant variability of age between the groups [4, 5], older patients [4], and different sex ratio [4] without CSF results [4]). Furthermore, the goals of the respective studies have led to a different kind of the classification of the patients. Consequently, those differences make comparison difficult between the studies; however, some results are convergent. The cognitive clinical presentation was predominant in our study as in Morena et al. as well as a low percentage of patients with CSF AD profile in Inguanzo (11%) and in our study (18.3%). In our study, patients in the motor group showed a significantly increased risk of developing a full clinical picture compared to the cognitive group. This faster clinical evolution is in accordance with a faster rate of progression for DLB patients with parkinsonian symptoms at onset and an increased risk of loss of autonomy in the motor group [4]. Altogether, those results suggest a more rapid diffusion of neuropathological lesions in the motor group. However, in our cohort, this result contrasts with the absence of a significant difference between the 3 groups regarding the median survival time before the occurrence of a poor prognostic event (death or loss of autonomy) suggesting that the full clinical picture could not be link to the global evolution leading to the death of the patient. In addition, the occurrence of the full clinical picture could be, for some patients, mainly in the motor group, explained by the occurrence of visual hallucinations induced by dopaminergic drugs.

Regarding the comparison with the other studies, the lack of significant differences in mortality or prognosis among the patient groups could be due to the way of classification of our groups. Specifically, previous literature has described possible pathophysiological connections between cognitive fluctuations and visual hallucinations [8], as well as associations between MRI patterns and distinct clinical profiles different from those used in our study [5]. Those associations could lead to different ways of the categorization of the patients. However, our goal was to be the closest as possible from daily clinical practice, meaning with the absence of specific brain MRI analysis and recording the initial symptoms with the patient and the family that have sometimes difficulties to note the fluctuations.

Similarly to previous studies [9], in this larger cohort with comparable 3 phenotype groups, we confirmed that patients with co-pathology (indicated by AD CSF biomarkers profile) were significantly associated with an increased instantaneous risk of poor prognosis. All those data suggest that cognitive deficit may lead to a faster occurrence of a poor prognosis event, and the presence of AD pathology clinically impacts the disease and may exacerbate the progression of DLB.

The strengths of the study are the number of patients, the multicentric methods including different expertise (memory clinics, Parkinson’s center), and the methodology based on daily clinical practice including comorbidities and variability in the clinical profile of patients. The limitations are the retrospective method, which may introduce potential biases, the lack of a sufficient number homogenous data including imaging, the lack of a specific psychiatric center due to the daily clinical practice in France, and the definition of preselected groups.

Altogether, the subclinical form of DLB is an emerging field with a current variable approach; however, in even various methodologies, those studies are able to demonstrate common results or trends according to the phenotype of the patients. Larger multicenter studies including therapeutic, imaging, and biological data and avoiding preselected groups with the use of principal component analysis are needed to provide more robust evidence on the impact of initial predominant symptoms on the clinical course and survival of DLB patients.

## Data Availability

Data and materials are available on request and in the limits of confidentiality for the patients.
